# Eczema compound patents’ herbal combination rules research based on complex system entropy clustering: A review

**DOI:** 10.1097/MD.0000000000032005

**Published:** 2022-12-09

**Authors:** Xujie Yang, Xiaohua Pei

**Affiliations:** a Hebei University of Chinese Medicine, Hebei, China; b Beijing University of Chinese Medicine, Xiamen Hospital, Fujian, China

**Keywords:** Complex system, eczema, entropy cluster, herbal compound, moisture and heat, prescription rule

## Abstract

The aim of this study is to investigate the herbal compound patents’ combination rules of eczema. Eczema herbal compound patents (oral drugs) from the past 29 years were collected, and a database was created. Complex system entropy clustering was used to analyze the prescription rule for eczema herbal compound patents. In total, 1039 eczema herbal compound patents, including 398 Chinese herbs, were identified. Complex system entropy clustering acquired high-frequency herbs, herbal couples, and core associations. Eczema herbal compound patents are known for clearing dampness and heat, dispelling poison, evacuating wind, and invigorating and cooling blood. By using complex system entropy clustering, herbal correlation of eczema herbal compound patents can be effectively analyzed.

## 1. Introduction

Eczema is a delayed hypersensitivity reaction caused by both internal and external factors. It is characterized by a diverse symmetrical rash, intense pruritus, and recurrence of a transudative skin inflammation. Based on skin lesion development, it is classified into 3 types: acute, subacute, and chronic. According to Western medicine, eczema is caused by an interaction between inner and outer body factors associated with type I and II hypersensitivity reactions. Although it is treated with antihistamines, glucocorticoids, and immunosuppressive, a satisfactory cure has not been achieved.

Complex system-based entropy clustering is the process of extracting valuable and potential knowledge from a large amount of data, also referred to as knowledge discovery in databases. It incorporates the latest database technologies, artificial intelligence, machine learning, statistics, knowledge engineering, object-oriented methods, information retrieval, high-performance computing, and data visualization. This is a multidisciplinary field of study. Complex system-based entropy clustering is an unsupervised pattern discovery algorithm extracting a combination with the largest amount of information from massive data by self-organization. This method is mainly suitable for highly discrete data, such as herbal compounds.^[1]^ It has been successfully applied in extracting clinical syndrome elements along with disease and syndrome. Since the traditional Chinese medical literature consists of discrete, continuous, mixed data, entropy methods based on the complex system make sense for analyzing prescription laws. This method has 2 significant benefits. It can qualitatively and quantitatively excavate the correlation between drugs and disease syndrome and symptoms drugs. Furthermore, it can excavate the core combination of doctors’ experience and excavate the herbal core combination hidden in the prescription compatibility, which the clinician has not valued.

The patent literature comprises novel, creative, and useful research results. In medicine, these results are of great significance for innovation and creation. Patent information mining can transform unordered data into valuable information, revealing any hidden messages in the patent. In this study, the oral herb prescription patents of eczema treatment from the past 29 years were selected. Herbal frequency, association, and composition compatibility regularity were investigated to guide clinical prescription and new drug development.

The herbal combination pattern determined by complex system entropy clustering could enhance the accuracy of clinical prescriptions, making selecting herbal core combinations easier and more accurate.

The traditional mutual information method has 2 limitations. Firstly, the calculation of mutual information is comparatively large; hence, the mutual information method does not consider the spatial position relationship of adjacent values. Secondly, when there is noise or the matching quality is not high, correct matching cannot be obtained. This study introduces the boundary mutual distance into the mutual information registration measure. By combining mutual information and mutual distance information, the new registration measure improves the mutual information registration measuring function, reducing the risk of the traditional maximum mutual information method falling into extremum and defining the accurate ratio of information about eczema patent compound composition.

**Figure 1.**
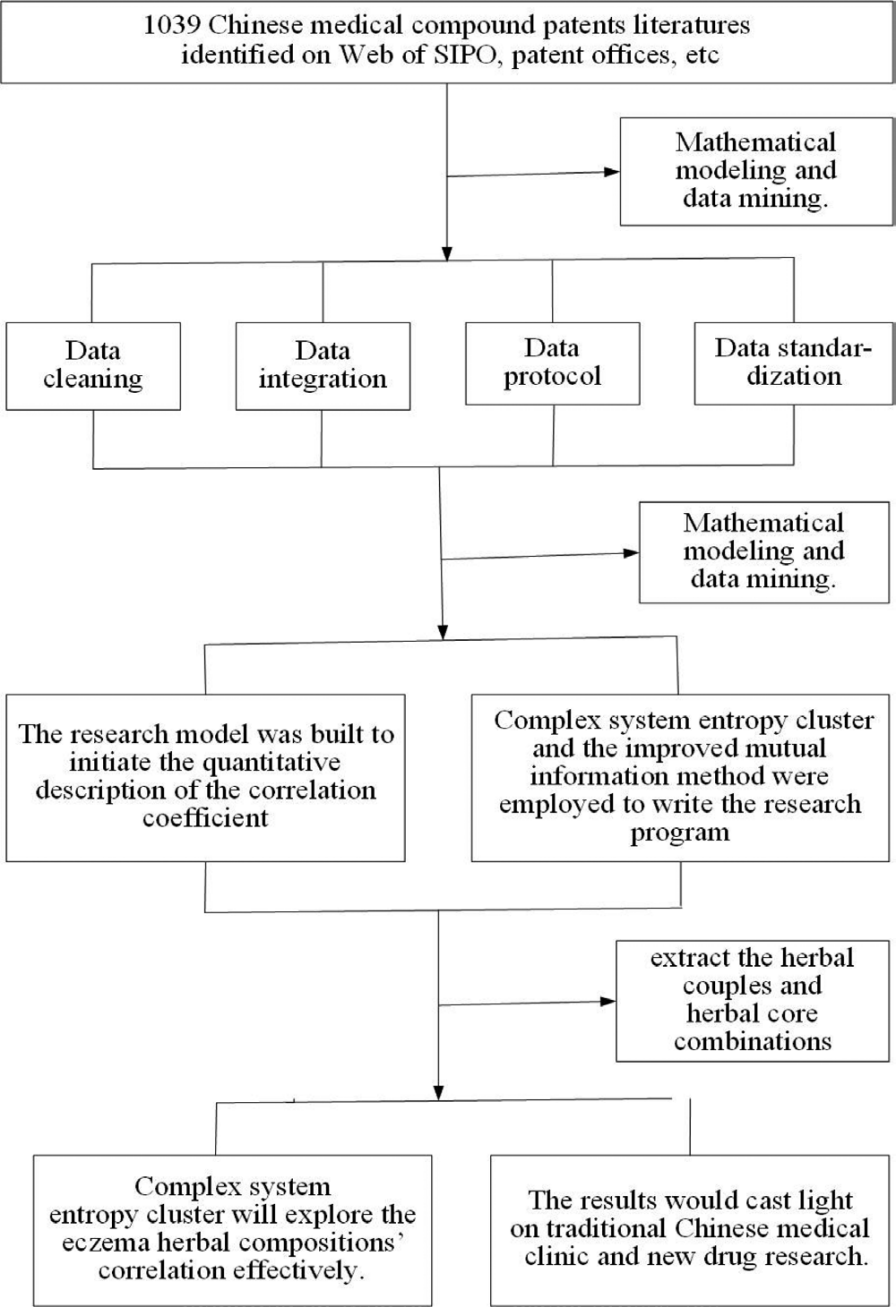
Algorithm of the research process.

On the basis of this study, future research will be based on the network pharmacology methods to obtain the main active ingredients of each drug pair and drug combination and their efficacy in treating eczema through TCMSP, DRAR-CPI, Genecards, OMIM, and other databases. Using Cytoscape 3.6.0 software, the active ingredient-potential target network will be constructed, and the interaction network of potential targets will be screened out along with the core targets, whose binding affinity with the active ingredients can be verified by molecular docking. An active ingredient-potential target-key pathway network will be constructed based on gene ontology and KEGG pathway enrichment analysis of potential targets. This study aims to explore the active ingredients, targets, pathways, and mechanisms of the drug pairs or drug combinations in the treatment of eczema (Fig. 1).

## 2. Methods

### 2.1. Collection and collation of eczema herbal compound patents

The literature collection covered the National Intellectual Property Office patent database, the Derwent World Patent Index database, the Multinational Patent Review Information Search System, The World Digital Library of Intellectual Property, traditional Chinese medicine enterprises, and related herbal intellectual property organizations. Eczema herbal compound patents with an application period between January 1993 and December 2021 were retrieved.

### 2.2. Database establishment

Epidata 3.1 was used to develop the information mining program. Each eligible eczema prescription patent herb was assigned a 2-valued variable.^[2]^ When the user clicks on the Chinese herbs involved in a prescription with the mouse, he automatically assigns them the value “1.” The remaining unclicked herbs were assigned the value “0” automatically. The database contained 1039 patents of compound prescriptions for eczema, including 398 commonly used Chinese herbs.

### 2.3. Mathematical modeling and data mining

Data mining was conducted using mathematical entropy modeling and screening out herbal pairs and core combinations with strong efficacy coherence. Complex system entropy clustering and the improved mutual information method were used to construct the research program and create the research model in order to initiate the quantitative description of the correlation coefficient of Chinese medicine. The eventual purpose was to extract the herbal couples and herbal core combinations.

Mathematical modeling was used to mine the eczema prescription patent. The most relevant core combinations of Chinese herbs were screened out by mathematical calculations, comprising core herb pairs and herb teams. The improved mutual information method, complex system entropy clustering method, and unsupervised entropy hierarchical clustering method were used for data analysis and precisely described the herbal correlation coefficient and core combination extraction. Here are the core formulas of the improved mutual information method and complex system entropy clustering method:


$$\begin{array}{l} \Delta \mu^{\prime }\left(X_{i},X_{j}\right)=\left\{ \begin{array}{l} \frac{H\left(X_{i}\right)+H\left(X_{j}\right)-H\left(X_{i},X_{j}\right)}{H\left(X_{j}\right)}P_{o}\left(i,j\right)\geq \delta \\ \frac{HX_{i}+HX_{j}-2HX_{i},X_{j}}{H\left(X_{j}\right)}P_{o}\left(i,j\right)<\delta \end{array}\right. \end{array}$$

Unsupervised hierarchical clustering can be described using the following formula:$MI^{\prime }\left(X,Y\right)=\left\{ \begin{array}{l} H\left(X\right)+H\left(Y\right)-H\left(X\cup Y\right)\;\;pro\left(X,Y\right)=\;0\\ H\left(X\right)+H\left(Y\right)-2*H\left(X\cup Y\right)\;\;pro\left(X,Y\right)>0 \end{array}\right.$

P_o_(i,j) signifies the frequency of positive occurrences of the 2 variables Xi and Xj, and b is a real number > 1 and a penalty coefficient. δ represents the threshold value. Selecting the appropriate threshold can separate the positive correlation from the negative correlation and avoid the interference caused by incorrect data. H(X) and H(Y) denote the information entropy of X and Y, respectively, whereas H(X ∪ Y) signifies joint entropy.

The above research process can also be expressed as follows:

µ(Xi, Xj) = C (Xi) + C (Xj)–C (Xi, Xj) = ∑i∑jq(Xi, Xj) lg$\frac{q\left(Xi,\;\;\;Xj\right)}{q\left(Xi\right)q\left(Xj\right)}$ Ⅰ

µ_ij=_$\frac{u\;\left(Xi,\;\;\;Xj\right)}{\sqrt{C\left(Xi\right)}\sqrt{C\left(Xj\right)}}$ Ⅱ

µ_ (Xi, Xj) = _$\left\{ \begin{array}{l} C\left(Xi\right)+C\;\left(Xj\right)-C\;\left(Xi,\;\;\;Xj\right)Qo\left(i,j\right)\geq \delta \\ C\left(Xi\right)+C\left(Xj\right)-bC\left(Xi,Xj\right)Qo\left(i,j\right)<\delta \end{array}\right.$Ⅲ

Δµ(Xi,Xj)=$\left\{ \begin{array}{l} \frac{C\left(Xi\right)+C\left(Xj\right)-C\left(Xi,Xj\right)}{C\left(Xj\right)}Qo\left(i,j\right)\geq \delta \\ \frac{C\left(Xi\right)+C\left(Xj\right)-C\left(Xi,Xj\right)}{C\left(Xj\right)}Qo\left(i,j\right)<\delta \end{array}\right.$ Ⅳ

In this formula, q(x_i_) and q(x_j_) represent the probability density function of variate X_i_, and the variates X_j_, C(X_i_), and C(X_j_) represent the entropy of variate X_i_ and variate X_j_. C(X_i_, X_j_) denotes the combination entropy of variate X_i_ and variate X_j_, and u_ij_ is a dimensionless quantity. When variate X_i_ and variate X_j_ are in perfect correlation, the value of variate u_ij_ is 1. When variate X_i_ and variate X_j_ are completely independent, the value of variate u_ij_ is 0. Generally, variate u_ij_ is valued between 0 and 1, whereas variate u_ij_ is equal to variate u_ji_. Q_o_(i, j) represents the positive frequency of variate X_i_ and variate X_j_, b is a penalty coefficient, and δ is the threshold value.

In total, 1039 prescriptions in the database were analyzed and screened using the methods mentioned above. A total of 124 Chinese herbs with high frequency and 45 regularly used drug pairs were obtained using the improved mutual information method, and 35 groups of core combinations were obtained using complex system entropy clustering.

## 3. Results

**Figure 2.**
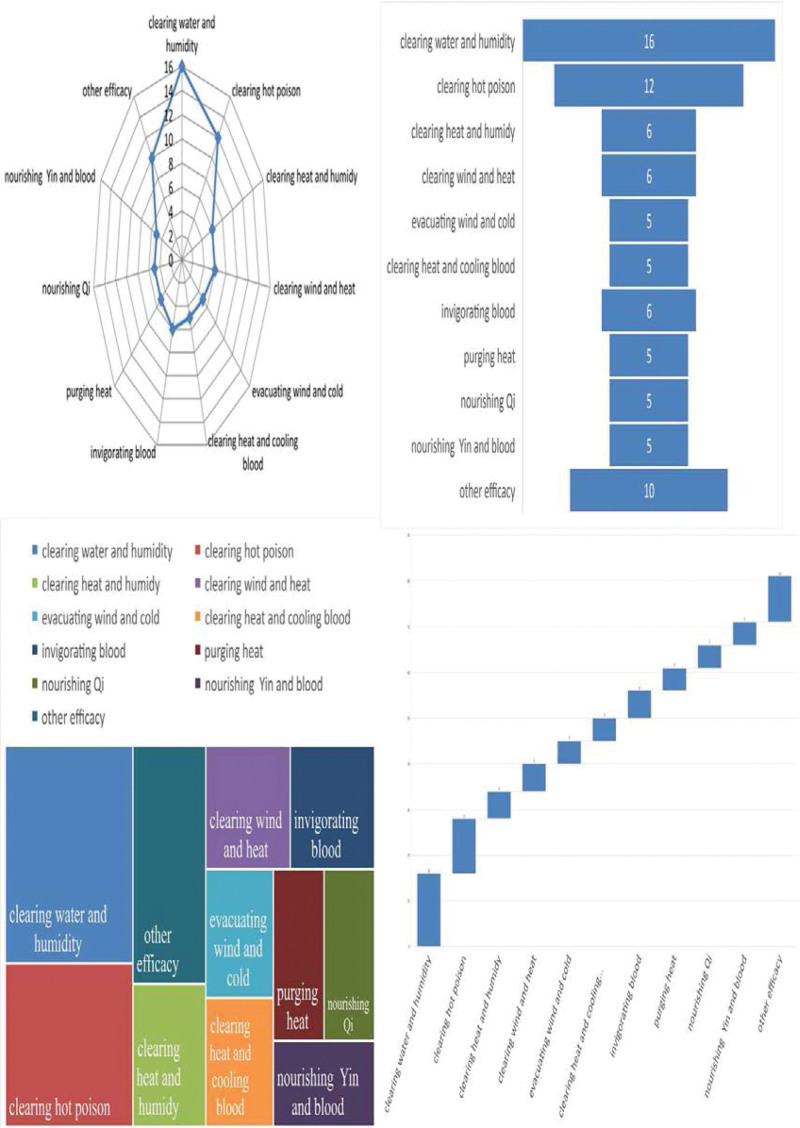
Frequency ratios of various functions’ herbs.

**Figure 3.**
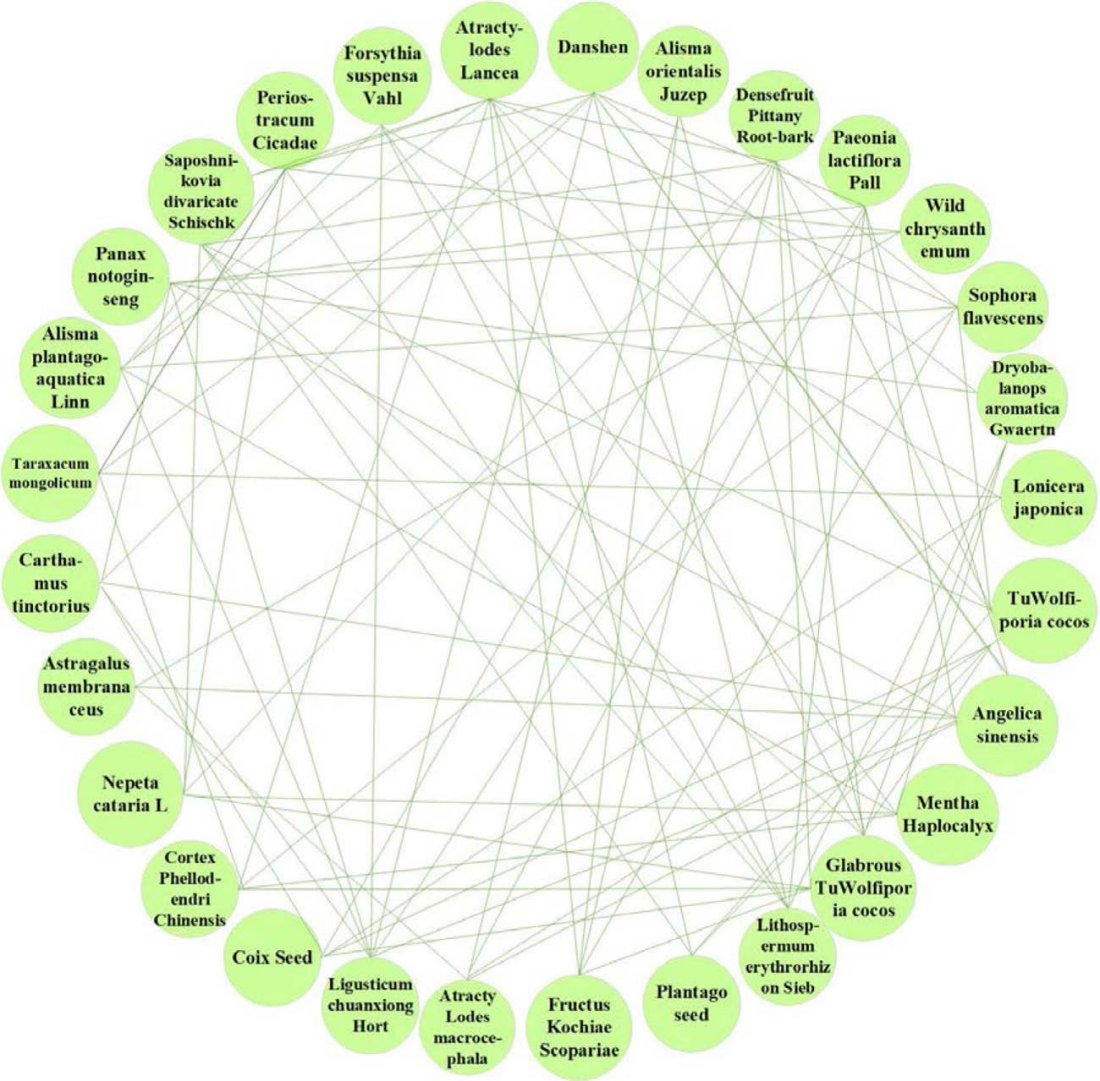
Herbal core combinations complex network.

**Figure 4.**
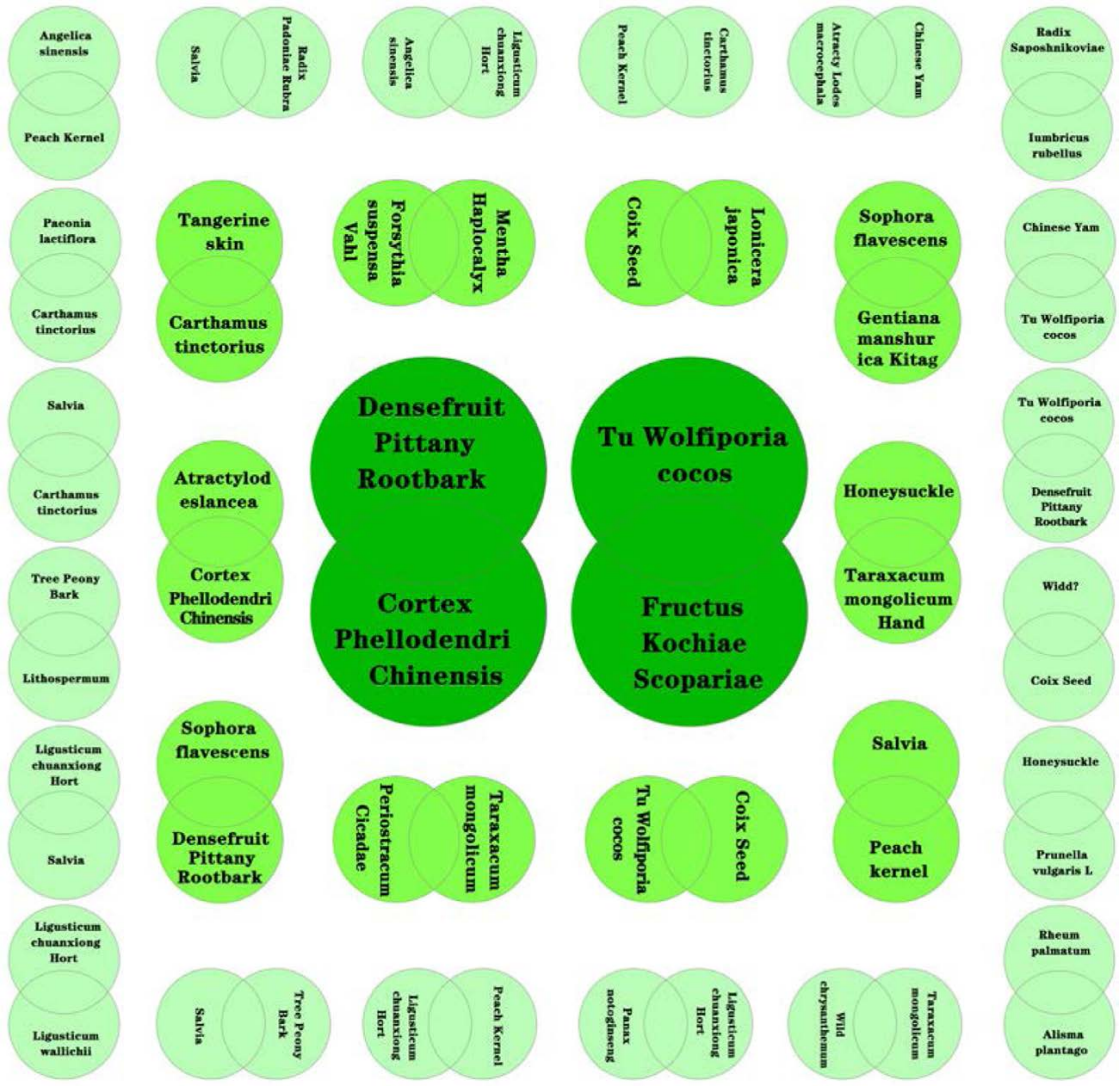
Drug pairs association.

**Figure 5.**
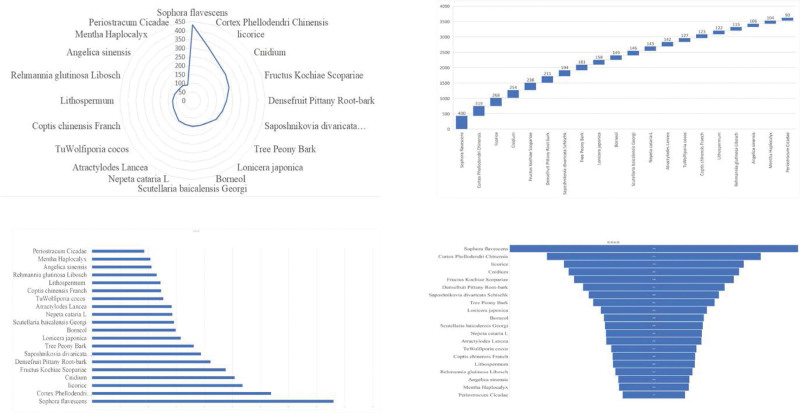
Frequency ratios of high frequency herbs.

**Figure 6.**
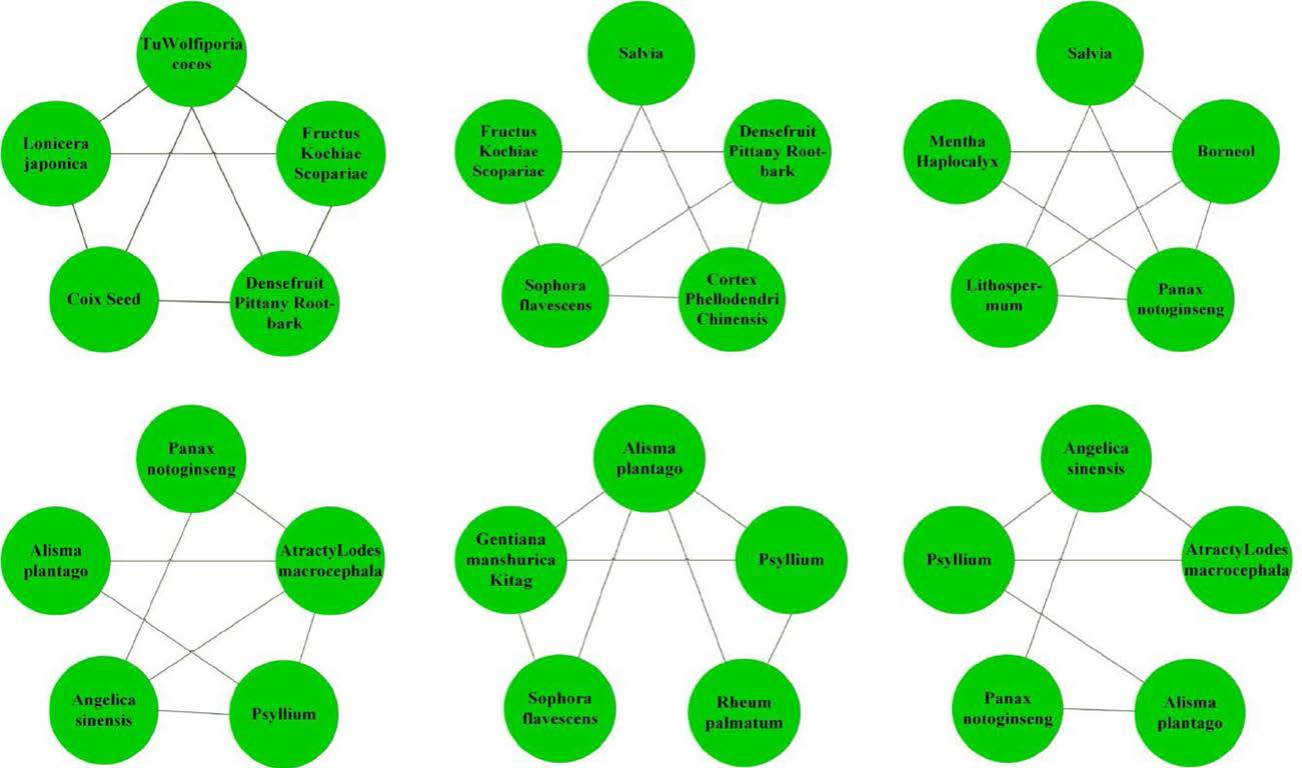
Herbal group complexation.

1. The high-frequency herbs, herbal couples, and herbal core combinations are included in Tables 1, 2, and 3, respectively.
2. The effects of high-frequency herbs were observed in heat elimination, dampness clearing, detoxification, blood cooling, evacuation of wind and heat, blood circulation, soothing liver, etc. (Figs. 2 and 5). Herbal couples are made up of 2 herbs that work together synergistically to achieve better results. The couple consists of 2 herbs with similar efficacy that play a therapeutic role with mutual assistance and 2 herbs with similar efficacy that are superimposed on each other (Table 4, Fig. 4). Herbal core combinations suggest that several herbs work together and appear together in most descriptions. These combinations are expressed as drug groups with the same or similar efficiency (Table 5, Figs. 3 and 6).

**Table 1:** High-frequency herbs.

No	Herb	Frequency	No	Herb	Frequency
1	Sophora flavescens	430	30	Rhus chinensis Mill	66
2	Cortex Phellodendri Chinensis	319	31	Plantago seed	66
3	Licorice	268	32	Alisma orientalis	65
4	Cnidium	254	33	Talcum	64
5	Fructus Kochiae Scopariae	238	34	Natural Indigo	62
6	Densefruit Pittany Root-bark	211	35	Forsythia suspensa	60
7	Saposhnikovia divaricata Schischk	194	36	Potassium alum	59
8	Tree Peony Bark	181	37	Carthamus tinctorius	58
9	Lonicera japonica	158	38	Artemisia argyi	57
10	Borneol	149	39	Stemonasessi	55
11	Scutellaria baicalensis Georgi	146	40	Astragalus membranaceus	52
12	Nepeta cataria L	143	41	Capillary Wormwood Herb	48
13	Atractylodes Lancea	142	42	Sanguisorba officinalis	48
14	Wolfiporia cocos	127	43	Xanthium sibiricum	48
15	Coptis chinensis Franch	123	44	Potassium alum	44
16	Lithospermum	122	45	Tangerine skin	42
17	Rehmannia glutinosa Libosch	115	46	Paeonia lactiflora	37
18	Angelica sinensis	106	47	Flos Chrysanthemi	37
19	Mentha Haplocalyx	104	48	Gardenia jasminoides Ellis	37
20	Periostracum Cicadae	93	49	Aloe vera	36
21	Gentiana manshurica Kitag	88	50	Rehmannia glutinosa	36
22	Taraxacum mongolicum	85	51	Patrinia Herb	36
23	Folium Isatidis	79	52	Scrophularia	36
24	Wild chrysanthemum	78	53	Prunella vulgaris L	32
25	Paeonia lactiflora	74	54	Spatholobus	30
26	Coix Seed	73	55	Panax notoginseng	30
27	Peppercorn	73	56	Viola yedoensis	30
28	Rheum palmatum	71	57	Ligusticum chuanxiong Hort	30
29	Calamine	69			

**Table 2:** Drug pair and its correlation coefficient.

Herbal pair		Correlation coefficient	Herbal pair		Correlation coefficient
Wolfiporia cocos	Fructus Kochiae Scopariae	0.18752	Salvia	Carthamus tinctorius	0.0986
Densefruit Pittany Root-bark	Cortex Phellodendri Chinensis	0.18214	Tree Peony Bark	Lithospermum	0.0965
Sophora flavescens	Gentiana manshurica Kitag	0.17915	Ligusticum chuanxiong Hort	Salvia	0.0912
Lonicera japonica	Taraxacum mongolicum	0.17652	Ligusticum chuanxiong Hort	Ligusticum wallichii	0.0881
Salvia	Peach kernel	0.17341	Salvia	Tree Peony Bark	0.0835
Coix Seed	Lonicera japonica	0.16842	Ligusticum chuanxiong Hort	Peach kernel	0.0797
Forsythia suspensa Vahl	Mentha Haplocalyx	0.16521	Panax notoginseng	Ligusticum chuanxiong Hort	0.0775
Periostracum Cicadae	Taraxacum mongolicum	0.16130	Wild chrysanthemum	Taraxacum mongolicum	0.0769
Tangerine skin	Carthamus tinctorius	0.15682	Rheum palmatum	Alisma plantago	0.0742
Atractylodes lancea	Cortex Phellodendri Chinensis	0.15321	Lonicera japonica	Coix Seed	0.0713
Sophora flavescens	Densefruit Pittany Root-bark	0.15102	Lonicera japonica	Wolfiporia cocos	0.06956
Wolfiporia cocos	Coix Seed	0.14654	Coptis chinensis Franch	Cortex Phellodendri Chinensis	0.06838
Radix Saposhnikoviae	Lumbricus	0.1432	Tree Peony Bark	Peach kernel	0.06569
Chinese Yam	Wolfiporia cocos	0.1395	Plantago seed	Alisma plantago	0.06393
Wolfiporia cocos	Densefruit Pittany Root-bark	0.1362	Lonicera japonica	Forsythia suspensa	0.06199
Wild chrysanthemum	Coix Seed	0.1341	Tangerine skin	Xanthium sibiricum	0.05875
Lonicera japonica	Prunella vulgaris L	0.1311	Salvia	Panax notoginseng	0.05703
Chinese Yam	AtractyLodes macrocephala	0.1297	Lonicera japonica	Forsythia suspensa	0.05632
Peach kernel	Carthamus tinctorius	0.1275	Rheum palmatum	Alisma plantago	0.05487
Ligusticum chuanxiong Hort	Angelica sinensis	0.1168	Mentha Haplocalyx	Lonicera japonica	0.05321
Radix Padoniae Rubra	Salvia	0.1142	Tree Peony Bark	Carthamus tinctorius	0.05223
Angelica sinensis	Peach kernel	0.1078	Salvia	Spatholobus	0.05187
Paeonia lactiflora	Carthamus tinctorius	0.1042			

**Table 3:** Herbal core combinations.

No	Herbal core combination				
1	Atractylodes Lancea	Densefruit Pittany Root-bark	Sophora flavescens	Wolfiporia cocos	Chinese Yam
2	Sophora flavescens	Atractylodes Lancea	Densefruit Pittany Root-bark	Fructus Kochiae Scopariae	Alisma Plantago
3	Wolfiporia cocos	Plantago seed	Alisma plantago	Atractylodes Lancea	Plantago seed
4	Nepeta cataria	Radix Saposhnikoviae	Lumbricus	Periostracum Cicadae	
5	Wolfiporia cocos	Coix Seed	Chinese Yam	AtractyLodes macrocephala	
6	Wolfiporia cocos	Fructus Kochiae Scopariae	Densefruit Pittany Root-bark	Coix Seed	
7	Sophora flavescens	Gentiana manshurica Kitag	Alisma Plantago	Plantago seed	
8	Atractylodes Lancea	Cortex Phellodendri Chinensis	Sophora flavescens	Fructus Kochiae Scopariae	
9	Tree Peony Bark	Lithospermum	Angelica sinensis	Panax notoginseng	
10	Salvia	Ligusticum chuanxiong Hort	Angelica sinensis	Paeonia lactiflora	
11	Ligusticum chuanxiong Hort	Angelica sinensis	Panax notoginseng	Paeonia lactiflora	
12	Panax notoginseng	Angelica sinensis	Carthamus tinctorius	Ligusticum chuanxiong Hort	
13	Nepeta cataria	Mentha Haplocalyx	Radix Saposhnikoviae	Forsythia suspensa	
14	Lonicera japonica	Forsythia suspensa	Lithospermum	Viola yedoensis	
15	Nepeta cataria	Fructus Kochiae Scopariae	Densefruit Pittany Root-bark	Radix Saposhnikoviae	
16	Atractylodes Lancea	Cortex Phellodendri Chinensis	Sophora flavescens	Alisma Plantago	
17	Tree Peony Bark	Salvia	Borneol	Lithospermum	
18	Lonicera japonica	Coptis chinensis Franch	Cortex Phellodendri Chinensis	Taraxacum mongolicum	
19	Astragalus Membranaceus	Angelica sinensis	Paeonia lactiflora	AtractyLodes macrocephala	
20	Tangerine skin	Wolfiporia cocos	Licorice		
21	Radix Saposhnikoviae	Periostracum Cicadae	Atractylodes Lancea		
22	Angelica sinensis	Ligusticum chuanxiong Hort	Paeonia lactiflora		
23	Angelica sinensis	Carthamus tinctorius	Lithospermum		
24	Panax notoginseng	Salvia	Borneol		
25	AtractyLodes Macrocephala	Alisma orientalis	Plantago seed		
26	Mentha Haplocalyx	Panax notoginseng	Borneol		
27	Periostracum Cicadae	Viola yedoensis	Lithospermum		
28	Angelica sinensis	Salvia	Taraxacum mongolicum		
29	Periostracum Cicadae	Taraxacum mongolicum	Wild chrysanthemum		
30	Carthamus tinctorius	Angelica sinensis	Salvia		
31	Paeonia lactiflora	Panax notoginseng	Lithospermum		
32	Coix Seed	Alisma orientalis	Fructus Kochiae Scopariae		
33	Densefruit Pittany Root-bark	Taraxacum mongolicum	Fructus Kochiae Scopariae		
34	Lonicera japonica	Taraxacum mongolicum	Lithospermum		
35	AtractyLodes Macrocephala	Wolfiporia cocos	Plantago seed		

**Table 4:** Herbal pair’s efficacy, pathological mechanism. Representative symptom.

Herbal pair		Efficacy	Pathological mechanism	Representative symptoms
TuWolfiporia cocos	Fructus Kochiae Scopariae	Clearing away heat and dampness, dispelling wind and relieving itching，Nourishing yin -essence and kidney	subacute or chronic eczema, Wind, dampness, and heat are stagnating, both Qi-essence and Yin-essence are deficient.	Generalized skin lesions, severe itching, skin erosion, fluid exudation, thin body, dizziness, and insomnia
TuWolfiporia cocos	Densefruit Pittany Root-bark			
TuWolfiporia cocos	Coix Seed			
Densefruit Pittany Root-bark	Cortex Phellodendri Chinensis	Clearing away heat and dampness, dispelling wind, purging fire, detoxifying.	Acute or subacute eczema, depression of dampness, heat, wind and poison	Severe itching, erosion into flakes, a lot of exudate
Sophora flavescens	Densefruit Pittany Root-bark			
Sophora flavescens	Gentiana manshurica Kitag	Clearing away heat and dampness, purging liver and gallbladder fire.	Acute or subacute eczema, depression of dampness and heat, insufficiency of liver and gallbladder function	Severe itching, erosion into flakes, a lot of exudate, depressed, easily angered
Lonicera japonica	Taraxacum mongolicum	Clearing away heat and dampness, purging fire and detoxifying	Acute eczema, stagnation of dampness, heat, fire and poison	Severe itching, redness, swelling, heat and pain, massive erosion and exudation
Wild chrysanthemum	Coix Seed Coix Seed			
Salvia	peach kernel	Activating and cooling blood, removing blood stasis, relieving pain	subacute or chronic eczema, blood heat and stasis	Pigmentation, rough surface, scaly, pain
Radix Padoniae Rubra	Salvia			
Angelica sinensis	peach kernel	Activating and nourishing blood, removing blood stasis.	The later stages of various types of eczema, Qi and blood stasis, insufficient blood and essence.	Prolonged eczema, lingering and difficult to heal, severe pain，Dull skin, dizziness and fatigue
Paeonia lactiflora	Carthamus tinctorius			
Ligusticum chuanxiong Hort	Angelica sinensis			
Coix Seed	Lonicera japonica	Clearing away heat and dampness, purging fire and detoxifying	Acute or subacute eczema, stagnation of dampness, heat, fire and poison	Redness, swelling, heat pain, erosion and exudation, severe itching
Forsythia suspensa Vahl	Mentha Haplocalyx	Clearing heat and detoxifies, dispersing knots, reducing swelling	Acute eczema, stagnation of heat, fire and poison	Lesion is red, swollen, hot, and painful
Periostracum Cicadae	Taraxacum mongolicum	Dispersing wind-heat, detoxifing, reducing swelling	Acute eczema, stagnation of wind, poison, heat or fire	Lesion is red, swollen, hot, and painful, intense itching.
Tangerine skin	Carthamus tinctorius	Regulating Qi and activating blood, drying dampness and removing blood stasis	chronic eczema, Qi stagnation, blood stasis, damp turbidity	Skin thickening, infiltration, brown-red pigmentation, rough surface, scaly
Atractylodes lancea	Cortex Phellodendri Chinensis	Clearing heat and drying dampness, invigorating spleen and invigorating Qi	chronic eczema, stagnation of dampness and heat, Qi deficiency	Eczema spread over multiple parts of the body, groggy feeling
Radix Saposhnikoviae	Lumbricus	Clearing away heat, dampness and wind, dredging collaterals	Acute or subacute eczema, stagnation of wind, dampness and heat	The itching rises and falls, gradually merges into pieces, pustules or scabs come into being.
Chinese Yam	TuWolfiporia cocos	Nourishing blood and Qi	In the later stage of various types of eczema, both Qi and blood are depleted.	The patient is weak and pale, papules, herpes or blisters rise and fall, the sore surface is eroded.
Lonicera japonica	Prunella vulgaris L	Clearing heat, drying dampness, detoxifying body, dissipating lumps.	Acute or subacute eczema, stagnation of dampness, heat, poison.	The sore is red, swollen and hot, sore surface erosion, severe itching and painful.
Chinese Yam	AtractyLodes macrocephala	Nourishing spleen and stomach, dispelling wind and dampness	Acute or subacute eczema, stagnation of wind and dampness, weak spleen and stomach	Large area of eczema, one after another, erosion and exudation of the sore surface, fatigue, lack of food and diarrhea
peach kernel	Carthamus tinctorius	Activating blood and removing blood stasis	Subacute or chronic eczema, stagnation of blood.	Eczema with severe pain, fixed sore spot, brown-red pigmentation, rough surface, dull complexion

**Table 5:** Herbal Group’s Efficacy, Pathological Mechanism. Representative Symptom.

Herbal Group	Efficacy	Pathological Mechanism	Representative Symptoms
Atractylodes lancea, Densefruit Pittany Root-bark, Sophora flavescens, TuWolfiporia cocos, Chinese Yam	Clearing heat and drying dampness, dispelling wind and detoxifying body, strengthening spleen and replenishing Qi, nourishing blood and Yin-essence	Subacute or chronic eczema, stagnation of heat, dampness, wind, poison, weak spleen and stomach, deficiency of blood, Qi-essence and Yin-essence	Eczema is recurring, one after another, infiltration of the affected area, severe itching, weak spleen and stomach without appetite, diarrhea, weakness, dull complexion
TuWolfiporia cocos, Coix Seed, Chinese Yam, AtractyLodes macrocephala			
Sophora flavescens, Atractylodes lancea, Densefruit Pittany Root-bark, Fructus Kochiae Scopariae, Alisma plantago	Clearing heat and drying dampness, dispelling wind and detoxifying body, strengthening spleen and replenishing Qi	All kinds of eczema, stagnation of heat, dampness, wind, poison, weak spleen and stomach	Eczema is recurring, one after another, infiltration of the affected area, severe itching, weak spleen and stomach without appetite, diarrhea
Atractylodes lancea, Cortex Phellodendri Chinensis, Sophora flavescens, Alisma plantago			
Atractylodes lancea,Cortex Phellodendri Chinensis, Sophora flavescens, Fructus Kochiae Scopariae			
TuWolfiporia cocos，Plantago seed，Alisma plantago，Atractylodes lancea	Clearing heat and drying dampness, kidney-tonifying and Promoting urination, strengthening spleen and replenishing Qi, nourishing blood and Yin-essence	Subacute or chronic eczema, stagnation of heat and dampness, weak spleen, stomach and kidney, insufficient Qi, blood and Yin- essence	Eczema is red, swollen, hot and painful, lesion erosion and exudation, obvious systemic symptoms, weak spleen and stomach without appetite, diarrhea, weakness, dull complexion, weak limbs, waist and knees, puffy body, difficulty urinating
Nepeta cataria， Radix Saposhnikoviae，Lumbricus，Periostracum Cicadae	Clearing heat and drying dampness, dispelling wind and soothing skin surface, dredging meridians, promoting the appearance of rash	Early stage of acute eczema, stagnation of wind, dampness and heat, meridian stasis	Eczema is characterized by numerous dense, miliary-sized papules, blisters, or vesicles, the rash base is light red, gradually coalesces into large area with intense itching
Nepeta cataria，Mentha Haplocalyx，Radix Saposhnikoviae，Forsythia suspensa	Dispelling wind and relieving muscle surface, clearing heat and detoxifying the body, reducing swelling and dissipating knots	Acute eczema, stagnation of wind, heat and poison	Eczema is characterized by numerous dense, miliary-sized papules, blisters, or vesicles, the rash base is red, gradually coalesces into large area with intense itching, swollen and painful lesion
Nepeta cataria，Fructus Kochiae Scopariae，Densefruit Pittany Root-bark，Radix Saposhnikoviae	Dispelling wind and relieving itching, clearing heat and drying dampness, dispelling rash and eliminating sores, detoxifying the body and relieving pain	Acute eczema, stagnation of wind, dampness, heat and poison	Eczema is characterized by numerous dense, miliary-sized papules, blisters, or vesicles, the rash base is light red, gradually coalesces into large area with itching, swollen and painful lesion. Sore surface erosion, fluid oozing
TuWolfiporia cocos，Fructus Kochiae Scopariae，Densefruit Pittany Root-bark，Coix Seed	Clearing heat and drying dampness, dispelling wind and detoxifying body, nourishing blood and Yin-essence	Subacute or chronic eczema, stagnation of wind, dampness, heat and poison, insufficient blood and Yin-essence	Eczema is recurring, one after another, infiltration of the affected area, severe itching, dull complexion
Sophora flavescens, Alisma plantago, Gentiana manshurica Kitag, Plantago seed	Clearing heat and drying dampness, promoting urine excretion, eliminating limbs’ swelling, Purging liver and gallbladder fire.	Acute or subacute eczema, stagnation of dampness and heat, lver and gallbladder fire stasis.	Liquid exudation on the sore surface, erosion, itching, painful urination, swelling of limbs, irritability, insomnia.
Tree Peony Bark, Lithospermum, Angelica sinensis, Panax notoginseng	Clearing heat and cooling blood, promoting blood circulation and removing blood stasis, detoxifying the body and reducing swelling, nourishing blood and relieving pain	Acute or subacute eczema, stagnation of heat and poison, blood stasis, blood deficiency, swelling pain	Eczema is red, swollen, hot and painful, brownish red pigmentation, rough and dull skin, lightheadedness
Salvia, Ligusticum chuanxiong Hort, Angelica sinensis, Paeonia lactiflora	Cooling blood and relieving pain, promoting blood circulation and removing blood stasis, nourishing blood and Yin-essence	Subacute or chronic eczema, stagnation of heat and blood, insufficient blood and Yin-essence	Eczema is red, swollen, hot and painful, brownish red pigmentation, rough and dull skin, lightheadedness, paled face
Ligusticum chuanxiong Hort, Angelica sinensis, Panax notoginseng, Paeonia lactiflora	Promoting blood circulation and removing blood stasis, reducing swelling and relieving pain, nourishing blood and Yin-essence	Various types of eczema, stagnation of blood, insufficient blood and Yin-essence	Swelling and pain in the affected area, brown-red pigmentation, swelling pain,rough surface, dull skin, lightheadedness, paled face, insomnia forgetfulness
Panax notoginseng, Panax notoginseng, Angelica sinensis, Carthamus tinctorius, Ligusticum chuanxiong Hort	Nourishing and activating blood, removing blood stasis, reducing swelling and relieving pain	Various types of eczema, stagnation of blood, insufficient blood	Swelling and pain in the affected area, brown-red pigmentation, rough surface, dull skin, lightheadedness
Lonicera japonica, Forsythia suspensa, Lithospermum, Viola yedoensis	Clearing heat and detoxifying the body, reducing swelling and dissipating knots, cooling blood and activating blood	Acute eczema, stagnation of heat, blood and poison	The skin lesions are dense papules, herpes or small blisters, bright red in color, gradually converging into pieces, the sore surface is hot and painful, and the swelling is obvious
Lonicera japonica, Coptis chinensis Franch, Cortex Phellodendri Chinensis, Taraxacum mongolicum	Clearing heat and dampness, purging fire and detoxifying the body, clearing liver and gallbladder fire, reducing swelling and dispersing knots	Acute eczema, stagnation of dampness, heat, fire and poison, fire stagnation in the liver and gallbladder	Eczema is manifested as papules, herpes or small blisters, which gradually merge into patches, with severe redness, swelling, heat and pain
Astragalus membranaceus, Angelica sinensis, Paeonia lactiflora, AtractyLodes macrocephala	Nourisheing Qi and Yin-essence, nourisheing and activating blood, eliminating dampness and strengthening spleen	In the late stage of subacute or chronic eczema, Qi, blood, Yin-essence are all insufficient	The disease course is very long, and it can’t heal for a long time, weakness, lack of food, diarrhea, dull complexion, insomnia and forgetfulness
Tree Peony Bark, Salvia, Borneol, Lithospermum	Clearing heat and cooling blood, promoting blood’s circulation and removing blood stasis, opening orifices and detoxifying the body	acute eczema, stagnation of heat, blood and poison, meridians blocked	Erosion and exudation, redness, swelling, heat, pain, severe itching, dizziness, red eyes, mouth sores, sore throat

## 4. Discussion

### 4.1. Acknowledge chinese medicine on eczema

Eczema is caused by the body’s weakness and the intrusion of wind, heat, and wet evil. It is divided into 3 types: acute, subacute, and chronic. Acute eczema mainly results from damp heat. The deficiency of the spleen system results in wet evil resistance, leading to subacute eczema. Chronic eczema occurs when the skin cannot be nourished due to long illnesses and blood loss. Acute eczema spreads throughout the body; chronic eczema mainly damages some parts of the body, while subacute eczema has symptoms between acute and chronic eczema.^[3]^

The 3 types coexist in the same period, but one type dominates in most cases. The 4 pathogenic factors (damp heat, spleen weakness, long illness, and blood loss) also affect the body. Therefore, the treatment of eczema by herbal prescription must consider the 4 pathogenic factors even while concentrating on one pathogenic factor. Clinically, eczema is always stratified into 2 major cure species: damp-heat siltation and skin undernutrition for blood weakness.

### 4.2. Analysis of high-frequency herbs and their active pharmaceutical ingredients for treating eczema

According to the findings, herbs that clear damp heat are widely used. The number of damp heat-clearing herbs is 32 in high-frequency herbs. Damp-heat siltation eczema is treated by removing dampness and heat. When the heat proportion is greater than dampness, clearing fire and detoxification play a crucial part. Spleen strengthening and clearing dampness play an important role when the dampness proportion is greater than the heat, whereas the dispelling of dampness by aromatic herbs will serve as adjuvant therapy. Blood weakness causes eczema, which is treated by nourishing the blood and eradicating other pathogenic factors. In most cases, numerous treatments are introduced, such as delighting the liver, dredging blood stasis, and eliminating wind evil.

In this research, the high-frequency Chinese herbs had excellent clinical effects on eczema. The use of *Sophora flavescen*s, the dried root of the leguminous plant *Sophora flavescens,* was the most frequent (Table 1). Its taste is extremely bitter, and its property is cold. It is therapeutic in the meridian systems of the heart, liver, stomach, large intestine, and bladder. It effectively clears heat and dampness, dispelling wind and arresting itch, killing parasites, and eliminating urine. It is always used in treating itchy skin, pustular sores, scabies, and leprosy. In eczema, it usually acts as a master herb with the maximum dose. According to traditional Chinese medicine theory, its effect of removing dampness and heat is achieved by acting on the mentioned organs. Alkaloids (matrine, oxymatrine, noxysophocarpine, sophoridine, I-somatrine, sophoranol, anagyrine, baptifoline, kuraridinol, kurarinol, neokurarinol, norkurarinol, and isokurarinone) are the primary chemical compounds of *S. flavescens* used in treating eczema.

The anti-allergy properties of m*atrine* can reduce the release of allergy mediators; therefore, it is used as an immunosuppressant. *Matrine* has an antibacterial effect both inside and outside the body. Its intensity inside the body is comparable with that of chloramphenicol, and the alcohol extract has an antitrichomoniasis effect outside the body.^[4]^ Furthermore, *matrine* and *oxymatrine* have inhibitory effects on immune function in mice. Intramuscular injection of oxymatrine can inhibit passive or active allergic skin reactions in rabbits and rats. Moreover, *oxymatrine* can significantly inhibit exudative inflammation induced by croton oil, carrageenan, and glacial acetic acid.

Traditionally, *Cortex Phellodendri Chinensis* herb is used to clear heat and wet turbidity. In addition to its ability to pyrolyze and detoxify, it tastes bitter and regulates the kidney, bladder, and large intestine. Its primary ingredients are berberine, phello dendrine, magnoflorine, jatrorrhizine, palmatine, menisperine, obacunone, etc. It has an antibacterial effect with a strong inhibitory effect in vitro on *Staphylococcus aureus, Bacillus anthracis*, *Helicobacter*, and hemolytic streptococcus, and its active antibacterial ingredient is berberine. Additionally, *Cortex Phellodendri Chinensis* has antifungal effects. The ethanol extract has strong antifungal activity against *Cryptococcus neoformans* and red psoriasis, and its effect is stronger than nystatin. Furthermore, it has an antitrichomonas effect, and its decoction has an inhibitory effect on *Trichomonas vaginalis*. Its decoction can increase the number of spleen plaque-forming cells in mice, inducing immune function.^[5]^

*Licorice* herb efficiently supplements spleen function, relieves pain, and reconciles herbal performance. It tastes sweet and has a distinct effect on the meridian systems of the heart, lung, spleen, and stomach. *Glycyrrhiza polysaccharide*, a macromolecular polysaccharide extracted from *licorice,* has a strong inhibitory effect on bacteria^[6]^ and can increase the activity of natural killer cells and antibody-dependent cell-mediated cytotoxicity both inside and outside the body. It can activate the lymphocyte proliferation of mice, selectively enhance the additive power and activity of helper T lymphocytes, and regulate multiple cytokine production and secretion.^[7]^ The anti-allergic effect of *licorice* is mostly due to glycyrrhizin, which can significantly prevent the release of histamine, acetylcholine, and other allergic compounds, thus inhibiting certain allergic reactions.^[8]^ Glycyrrhizin can considerably reduce the expression level of inflammatory factors, the activity of colon pulp peroxidase, and lipid-protein denaturation, and organ damage due to active oxidation substances.^[9]^

*Saposhnikovia divaricata Schischk* removes evil (wind and dampness evil) from the body surface and relieves pain. It has an evident effect on the meridian systems of the bladder, liver, and spleen. Its decoction significantly increases the pain threshold of mice and has a sedative and analgesic effect.^[10,11]^ Moreover, its volatile oil has an analgesic effect in each dose group. The analgesic and sedative effects of *S. divaricata Schischk* in eczema treatment are the results of synergy among various chemical components, such as color ketone, coumarin, polyacetylene, volatile oil, etc.^[12,13]^

### 4.3. Herbal pairs and combinations are simple and effective in treating eczema

Eczema can be effectively treated with herbal combinations and pairs. The findings of this study have revealed that the herbal pairs and their core combinations for the treatment and prevention of eczema are simple and basic traditional Chinese medical compatibility, including spleen strengthening, hot dampness clearing, blood circulation, cooling, detoxification, inducing resuscitation, attacking evil, dispelling wind, etc.

A drug pair is a combination of 2 herbs, which is the most basic form of an herbal compound. With reasonable collocation, it can improve or change the efficiency of monoherbs and guide the action orientation of monoherbs with multiple functions. In this research, some herbal pairs with the same efficiency played strong roles with 2-herb function superposition *(Wolfiporia cocos + Fructus Kochiae Scopariae, Cortex Dictamni + Cortex Phellodendri Chinensis, S. flavescens + Gentiana scabra Bunge, Lonicera japonica + Taraxacum mongolicum + Peach kernel*). Some other herbal pairs with different efficiency played a therapeutic role under the 2-herb mutual help (*Coix Seed + Lonicera japonica, Forsythia suspensa (Thunb) Vahl + Menthahaplocalyx, Periostracum Cicadae + Taraxacum mongolicum, Tangerine skin + Carthamus tinctorius L).*

There is a 42.8% inhibition rate of *Nepeta cataria’*s alcohol extract of pruritus. The permeability of the abdominal capillaries significantly decreases, which demonstrates significant anti-inflammatory effects.^[14,15]^ The effective part of *S. divaricata Schischk* has an evident anti-inflammatory effect.^[16]^ *Lumbricus* has antihistamine and antimetamorphosis properties.^[17]^ *Periostracum cicadae* can inhibit the release of allergic compounds by stabilizing mast cell granulation.^[18]^ As a result, the combination of *Nepeta cataria, S. divaricata Schischk, Lumbricus,* and *P. cicadae* can have powerful antiallergic and anti-inflammatory properties.

*Atractylodes Lancea (Thunb.) DC* and *Cortex Dictamni* have anti-inflammatory properties.^[19,20]^ *Oxidizing matrine* inhibits macrophage phagocytosis and prevents the release of histamine for mast cell degranulation.^[21]^ *Poria* has a strong inhibitory effect on cellular as well as humoral immunity.^[22,23]^ Water extract from *Wolfiporia cocos* can prevent mice contact with dermatitis and toe inflammation.^[24,25]^ Therefore, the combination of *Atractylodes Lancea (Thunb) DC, S. flavescens, Cortex Dictamni, and Wolfiporia cocos* can treat eczema with an anti-inflammatory and immunization effects.

Herbal pairs and core combinations used in this experiment were taken from an ancient classical compound, *Ermiao pill (Atractylodes Lancea + Cortex Phellodendri Chinensis*), created in the last Yuan Dynasty. In hot and humid immersions, it can remove dampness and heat. *Xiaofeng powder (S divaricata Schischk + Periostracum Cicadae + Atractylodes Lancea*) was created in *Ming·Waike Zhengzong*, which has antipyretic, dehumidifying, wind evil-removing, and blood-nourishing. It can treat eczema with several symptoms, such as intense itching, obvious heat, and severe eczema all over the body, and with 3 kinds of evil, such as heat, humidity, and wind.

The *Shenling Baizhu powder (Wolfiporia cocos + Coix Seed + Chinese Yam + AtractyLodes macrocephala*) was created in *Song·Taiping Huimin Heji Jufang.* It strengthens the spleen, promotes Qi function, and eliminates humidity. If eczema patients have spleen deficiency symptoms, such as thinning, pathological yellow complexion, fatigue, and stool accompanied by indigestible food can benefit from *Shenling Baizhu powder*. Additionally, *Erchen pill (Tangerine skin + Wolfiporia cocos + Licorice*) was created in *Song·Taiping Huimin Heji Jufang.* As well as clearing dampness and phlegm, it coordinates stomach and Qi functions. It is always used to treat eczema patients with phlegm wet symptoms, such as white phlegm, chest, and abdomen bulging, heavy limbs, white tongue coating, etc.

## 5. Conclusion

The findings of this study confirmed the following: the main pathogen of eczema is wet evil condensed in the body; therefore, the elimination of dampness is vital for its treatment. Blood and Qi may sludge in the body if wet evil deposits remain in the body for an extended period of time. The sludging of Qi and blood must transform into heat evil. Therefore, hot dampness also plays an important role in eczema pathogenesis.

Spleens and stomachs play an important role in the immune system, so the weakness of either will adversely affect immune function. Therefore, although eczema manifests in the skin, the source of the disease is in the spleen and stomach. The deficiency of the spleen (soil of the 5 elements) deteriorates the Qi operation mechanism, and the evil of humidity and heat is embodied. Having humidity and heat makes it easy for wind evil to attack. Wind, humidity, and heat result in rashing. Therefore, it is necessary to pay attention to the nourishment of the spleen (soil) to restrict humidity and heat. Some cases require removing evil (heat, dampness, and wind) as the main treatment, with strengthening the spleen and stomach can be used as an adjunctive treatment. In others, strengthening the spleen and stomach plays a major role in dissipating evil. In most cases, both the internal and external treatments are simultaneously applied.

Based on these results, network pharmacology will be used to discover each drug pair (combination)’s main active ingredients and their efficacy in treating eczema, and to investigate their interactions. Key pathways were identified using molecular docking methods to verify their binding affinity with active ingredients, which enabled comprehensive and systematic exploration of active ingredients, targets, pathways, and action mechanisms of drug pairs or combinations in the eczema treatment.

## Acknowledgments

Thanks for the financial support provided by the National Natural Science Foundation, Hebei Natural Science Foundation.

## Author contributions

This work was done by Yang Xujie and Pei Xiaohua.

**Conceptualization:** Xujie Yang¸ Xiaohua Pei.

**Data curation:** Xujie Yang¸ Xiaohua Pei.

**Formal analysis:** Xujie Yang¸ Xiaohua Pei.

**Investigation:** Xujie Yang

**Methodology:** Xujie Yang¸ Xiaohua Pei.

**Project administration:** Xujie Yang.

**Resources:** Xiaohua Pei.
